# A missense mutation in *Muc2* promotes gut microbiome and metabolome-dependent colitis-associated tumorigenesis

**DOI:** 10.1172/JCI196712

**Published:** 2025-11-06

**Authors:** Giulio Verna, Stefania De Santis, Bianca N. Islam, Eduardo M. Sommella, Danilo Licastro, Liangliang Zhang, Fabiano De Almeida Celio, Emily N. Miller, Fabrizio Merciai, Vicky Caponigro, Wei Xin, Pietro Campiglia, Theresa T. Pizarro, Marcello Chieppa, Fabio Cominelli

**Affiliations:** 1 Department of Medicine, Digestive Health Research Institute, and; 2 Department of Pathology, Case Western Reserve University (CWRU) School of Medicine, Cleveland, Ohio, USA.; 3 Department of Pharmacy, University of Salerno, Fisciano, Italy.; 4 AREA Science Park, Padriciano, Trieste, Italy.; 5 Department of Population and Quantitative Health Sciences, CWRU School of Medicine, Cleveland, Ohio, USA.; 6 Department of Pathology, University of South Alabama, Mobile, Alabama, USA.; 7 Department of Experimental Medicine (DiMeS), University of Salento, Lecce, Italy.

**Keywords:** Gastroenterology, Inflammation, Colorectal cancer, Microbiome

## Abstract

Colitis-associated cancer (CAC) arises from a complex interplay between host and environmental factors. In this report, we investigated the role of the gut microbiome using Winnie mice, an ulcerative colitis–like (UC-like) model with a missense mutation in the *Muc2* gene. Upon rederivation from a conventional (CONV) to a specific pathogen–free (SPF) facility, Winnie mice developed severe colitis and, notably, spontaneous CAC that progressively worsened over time. In contrast, CONV Winnie mice showed only mild colitis but no tumorigenesis. By comparison, when re-derived into germ-free (GF) conditions, SPF Winnie mice were protected from colitis and colon tumors, indicating an essential role for the gut microbiome in the development of CAC in these mice. Using shotgun metagenomics, metabolomics, and lipidomics, we identified a distinct proinflammatory microbial and metabolic signature that potentially drives the transition from colitis to CAC. Using either SPF Winnie or WT (Bl/6) donors, fecal microbiota transplantation (FMT) into GF Winnie recipients demonstrated that, while colitis developed regardless of the donor, only FM from SPF Winnie donors resulted in CAC in recipient mice. Our studies present a relevant model of CAC, providing strong evidence that the microbiome plays a key role in its pathogenesis, thus challenging the concept of colon cancer as a strictly nontransmissible disease.

## Abstract 2

This study reports a novel model of colitis-associated cancer and provides further evidence that the microbiome plays a key role in the pathogenesis of CAC.

## Graphical abstract

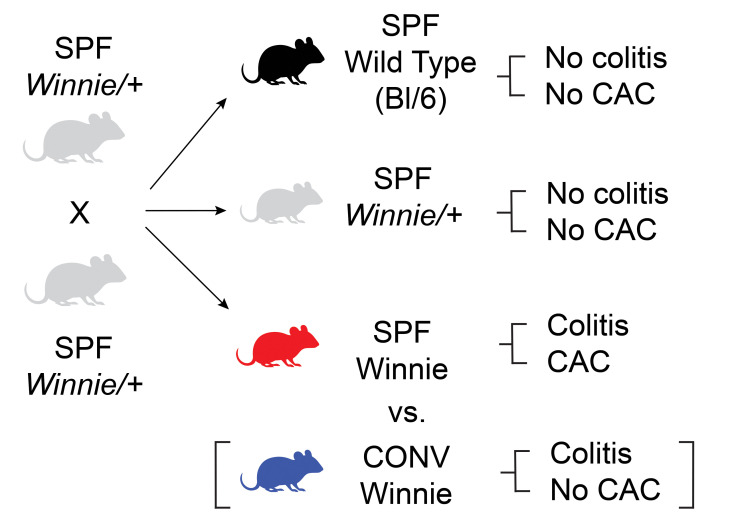

## Introduction

The pathogenesis of colitis-associated cancer (CAC) is a multifaceted process driven by chronic inflammation, genetic and epigenetic changes, dysregulated cell proliferation, oxidative stress, and alterations in the gut microbiome. A significant risk factor for CAC is ulcerative colitis (UC), characterized by persistent inflammation of the colon and rectum, and whose prevalence has been rising, particularly in western and newly industrialized countries (1). Patients with UC with longstanding and relapsing inflammation face an approximately 2- to 3-fold increased risk of developing colorectal cancer (CRC) (2). Despite advancements in surveillance and therapy, CAC remains a high-risk condition with increased mortality, often requiring colectomy as a last therapeutic option (3).

Although the interface between chronic inflammation and carcinogenesis is an area of active investigation, the precise mechanism(s) by which some, but not all, patients with UC progress to CAC remain unknown. This complexity arises from the challenge of understanding how multiple contributing factors interact, as they are often studied independently rather than comprehensively. Emerging evidence highlights a critical role for the gut microbiome and its metabolic byproducts in modulating CAC development (4, 5), suggesting that microbial interactions are essential among the multiple potential drivers of CAC. The gut microbiome, a complex and dynamic ecosystem, influences intestinal homeostasis by producing microbe-derived metabolites that modulate inflammation, immune function, and tumorigenesis (6). Dysbiosis, an imbalance in microbial composition, has been implicated in promoting a procarcinogenic environment through mechanisms such as the production of genotoxic compounds, disruption of epithelial integrity, and induction of chronic inflammation (7–9). Several experimental conditions demonstrate a decrease in colonic tumorigenesis under germ-free (GF) conditions, regardless of the nature of the trigger, whether genetic or chemically induced (10, 11). Thus, strategies including fecal microbiota transplantation (FMT), probiotics, prebiotics, synbiotics, postbiotics, and dietary modifications have been explored to enhance the effectiveness of immunotherapy by modulating the gut microbiome (12). Furthermore, the gut microbiome has been identified as both a biomarker and a modulator of antitumor immunotherapy outcomes (13, 14). Indeed, combining modulation of the gut microbiome with chemotherapy has been shown to enhance treatment efficacy in patients with CRC, suggesting that a synergistic approach to therapy may be more beneficial for these patients (15).

Interestingly, a recent finding suggests that alterations in both the gut microbiome and its metabolic products can influence the inflammatory environment, potentially contributing to the development of CAC (16). Additionally, metabolic pathway alterations may play a dual role in the pathogenesis of CAC. On one hand, amino acid metabolism and bile acid biosynthesis have been implicated in driving the inflammation-to-carcinoma progression characteristic of CAC (17). On the other hand, short-chain fatty acids (SCFAs), including acetate, propionate, and butyrate, have been shown to have an inverse correlation with inflammation, suggesting a potential protective effect against both chronic inflammation and CRC development (13). Several studies also demonstrated that lipids play a key role in CAC by influencing inflammation, immune responses, and tumor progression. Proinflammatory lipid mediators regulate inflammatory pathways associated with cancer development (18) and are present in colon tumors, indicating their influence on the tumor microenvironment (19). Additionally, dysregulated lipid metabolism promotes CAC progression by disrupting antitumor immune responses (20). Overall, these findings suggest that CAC develops through a multifaceted, intricate process that also involves the gut microbiome and its related metabolome/lipidome.

To gain deeper insights into the histopathologic and morphologic changes in CAC triggered by various factors and explore potential treatments, different murine models have been developed, including chemically induced, spontaneous, genetically modified, and adoptive transfer models (5). While these models have provided key insights into UC pathogenesis, replicating the full complexity of the human disease remains a significant challenge. Notably, the Winnie mouse strain has emerged as a promising model of UC-like colitis, closely mirroring the disease’s clinical and histopathological characteristics. Its ability to develop spontaneous colitis due to a point mutation in the *Muc2* gene, leading to epithelial barrier dysfunction, highlights the critical role of barrier damage in triggering inflammation, making it a valuable tool for studying UC progression and potential therapy (21, 22). Unlike chemically or genetically induced colitis models, Winnie mice naturally exhibit complex interactions among immune, epithelial, and neuronal cell populations and colonic microbiota, closely mirroring the complexity of human UC. Additionally, their spontaneous, fully penetrant phenotype, with disease manifesting as early as 4 weeks of age, provides a significant advantage over IL-10–KO and MDR1α-KO mice, in which colitis results from genetic deletion and is not consistently present in all individuals (5). Notably, before this study, despite exhibiting chronic and progressive inflammation in the large intestine, Winnie mice, to our knowledge, had never been reported to develop spontaneous CAC unless crossed with *APC^Min/+^* mice (23).

With the intent to gain a comprehensive perspective on the effect of the intestinal microbiota and associated metabolome/lipidome in colitis-prone Winnie mice, we re-derived Winnie mice, originally in conventional (CONV Winnie mice) housing (5, 22), into a specific pathogen–free (SPF) facility (SPF Winnie mice). Unlike what we previously published on CONV Winnie mice (23), we observed a more severe colitis in SPF Winnie mice, even without crossing them with *APC^Min/+^* mice, as we did in the prior study, and, surprisingly, an early onset of colonic tumorigenesis (at 4 weeks of age), with the extent and progression of disease increasing over time (up to 20 weeks of age). Using shotgun metagenomics and untargeted metabolomic and lipidomic approaches, we characterized the omics profiles of the 2 colonies (i.e., SPF Winnie and parental tumor-free CONV Winnie), revealing a microenvironment enriched in proinflammatory microbes, metabolites, and lipids that may be critical in transitioning from colitis to CAC in the SPF Winnie mice. Finally, to confirm the necessity of gut microbiota and the related metabolome/lipidome in CAC pathogenesis in SPF Winnie mice, we performed FMT using either SPF Winnie or WT littermate control (Bl/6) mice as donors and GF Winnie mice as recipients. Our data showed that colitis was similarly observed in GF Winnie recipients, independent of the donor feces. At the same time, only the transfer of SPF Winnie fecal material induced inflammation-associated colonic tumors, underscoring the importance — and perhaps the existence — of a specific CAC-predisposing gut microbiome milieu in SPF Winnie mice. Therefore, in the present study, we report what we believe to be a previously undescribed CAC model dependent on a nontumor-associated genetic mutation, combined with a unique intestinal microbiome and metabolome. We provide convincing evidence that the composition and function of the gut microbiome play a nonredundant role in promoting colonic tumorigenesis through FMT into GF recipients harboring a genetic factor, such as *Muc2*, involved in regulating gut homeostasis and colonic inflammation.

## Results

### SPF Winnie mice show a protumorigenic phenotype compared with CONV Winnie mice.

**Figure 1:**
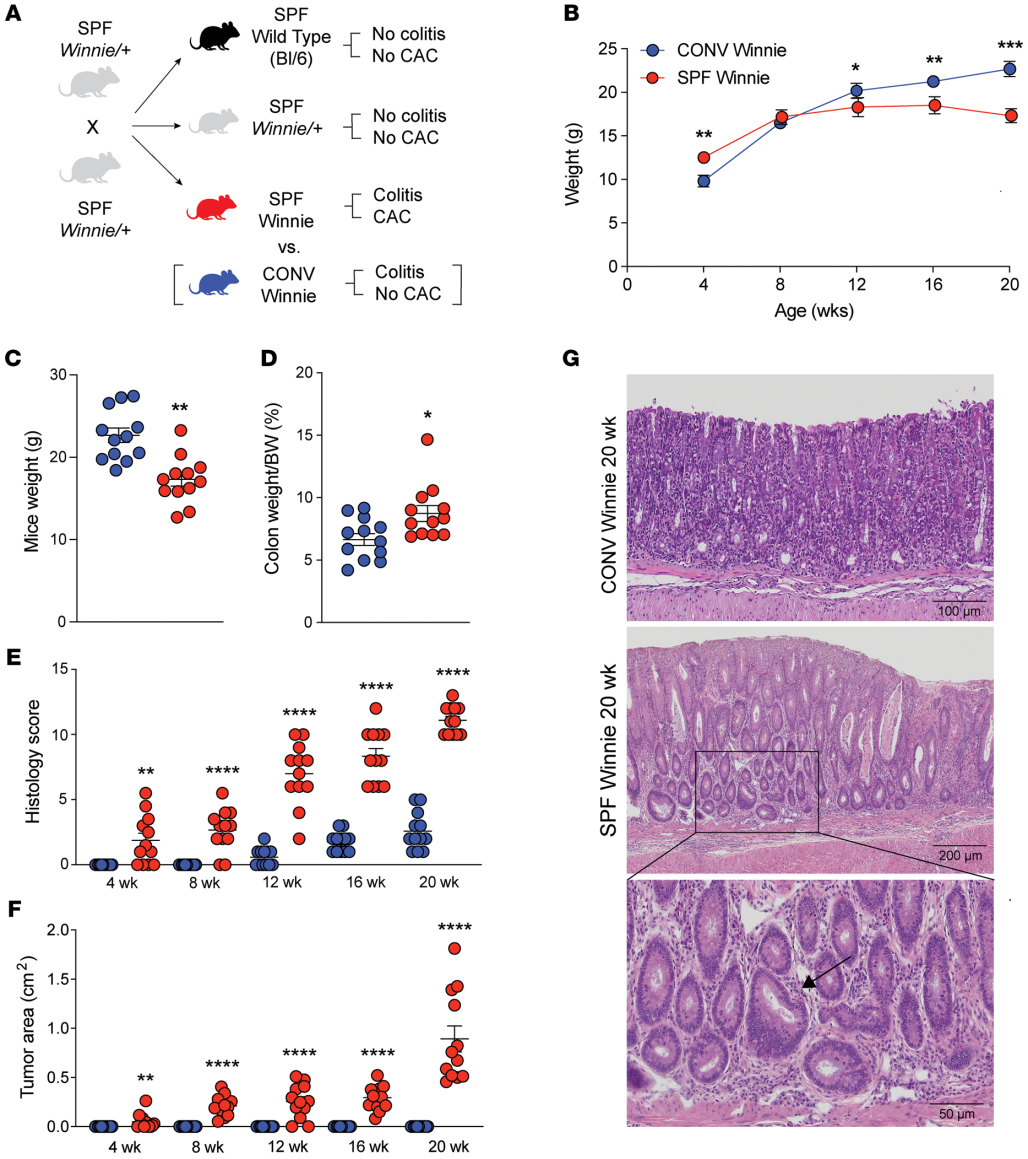
Increased disease severity and emergence of colonic tumors in SPF- versus CONV-raised Winnie mice. (**A**) Breeding strategy used to generate Winnie mice and their littermate controls (Bl/6) in SPF and CONV facilities, showing the specific phenotype of SPF Winnie, Winnie/+, and WT (WT Bl/6) mice. (**B**) Weight curves of SPF vs. CONV Winnie mice measured every 4 weeks until sacrifice at 20 weeks. (**C**–**G**) Necroscopic and histological evaluations of colonic inflammation and tumorigenesis comparing SPF versus CONV Winnie colons. (**C**) Weight and (**D**) colon weight/BW ratio percentage of experimental mice measured at the time of sacrifice. Progression of (**E**) disease severity over time as evaluated by a gastrointestinal (GI) pathologist blinded to the H&E-stained slides of colon samples, using an established scoring system. (**F**) Tumor area over time was calculated by Image J using a necropsy image of the colon taken at the time of sacrifice. (**G**) Representative H&E-stained images of colonic tissue from CONV (upper panel) and SPF (lower panel) Winnie mice at the time of sacrifice. Scale bars: 50 μm, 100 μm, and 200 μm. Original magnification, ×10 and ×20 (inset highlights dysplastic crypt [arrow]). Data presented in the dot plots are expressed as the mean ± SEM. *P < 0.05, **P < 0.005, ***P < 0005, and ****P < 0.0001, by unpaired 2-tailed *t* test or Mann-Whitney *U* test. *n* =12 for each experimental group.

**Figure 2:**
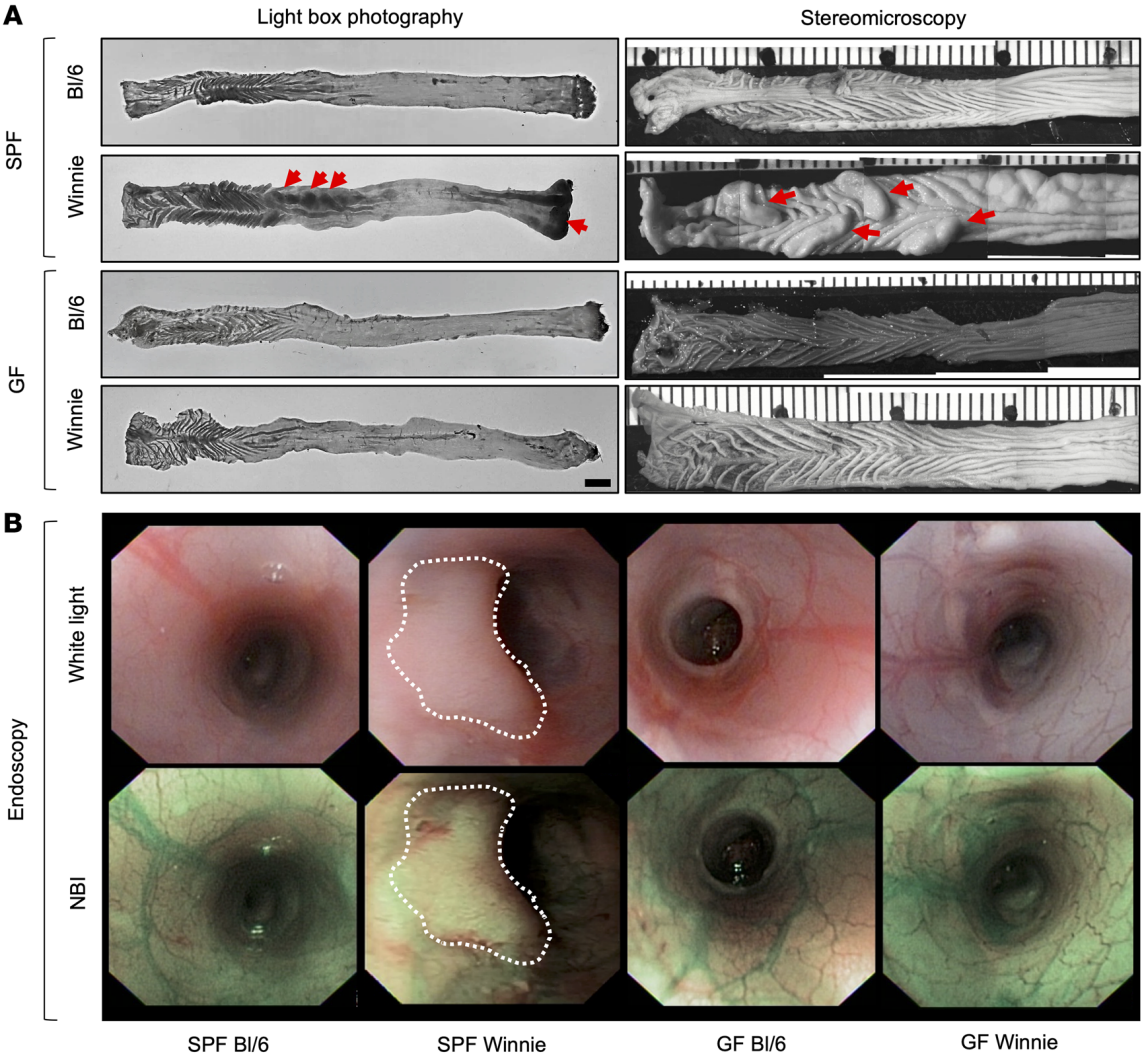
Development of colon tumors in SPF Winnie mice is dependent on the presence of gut microbiota. (**A**) Representative light box images (left panels) and stereomicroscopic images (right panels) of fresh and fixed, respectively, 20-week-old SPF and GF WT (Bl/6) and Winnie colons. (**B**) Representative endoscopic images of colons of 20-week-old SPF (left panels) and GF (right panels) Bl/6 and Winnie mice using white light (upper panels) and narrow band imaging (NBI) (lower panels). The white-dotted outline highlights the representative tumor area.

Winnie mice from a CONV facility in Italy were re-derived to a SPF facility in the United States. Heterozygotic Winnie mice (Winnie/+) were used for breeding to obtain Winnie mice and their littermate WT controls (WT, Bl/6 mice) from the same breeding. SPF Winnie mice developed a chronic colitis phenotype and CAC that increased in severity over time (Figure 1A). By comparison, SPF Winnie/+ mice and their WT (Bl/6) littermates had no macroscopic signs of colitis and/or CAC at any of the time points, with histological scores of 0, both when cohoused and when separated. Weight comparisons between the original CONV and the new SPF colonies from weaning (4 weeks) to sacrifice (20 weeks) revealed significant differences (Figure 1B). While SPF Winnie mice were initially heavier at 4 weeks of age, their growth stalled around 8 weeks of age, whereas CONV Winnie mice continued gaining weight (Figure 1B). By 12, 16, and 20 weeks, the CONV Winnie mice had significantly higher BWs, likely due to a worsening disease phenotype in the SPF Winnie mice (Figure 1, B and C). Despite no differences in colon length at sacrifice (data not shown), the SPF Winnie mice had a higher colon weight/BW ratio (Figure 1D), indicating colon-specific pathology. In line with this observation, a time-course analysis of H&E-stained colon showed an increase in the histology score over time in SPF versus CONV Winnie mice (Figure 1E), with only mild inflammation even at later time points (Figure 1E). In line with the inflammatory score, tumor area borders progressively expanded over time, even at early time points (4 weeks) (Figure 1F). Unlike the previously characterized Winnie-*APC^Min/+^* model (23), tumors in SPF Winnie mice were specifically located in the proximal and medial colon, as indicated by the light box and stereomicroscopic images (Figure 2A, left and right panels, respectively). Conversely, SPF Winnie/+ mice and Bl/6 littermates obtained from the same SPF-Winnie breeders did not show tumors or signs of inflammation at any time points when cohoused and separated. Data collected over time demonstrate that GF Winnie mice and their Bl/6 counterparts did not develop tumors or inflammatory phenotypes (Figure 2, A and B, respectively). Similar to the anatomical characteristics of human CAC, endoscopic observations confirmed the presence of neoformations protruding into the colon lumen of SPF Winnie mice (Figure 2B, upper row, white-dotted area) located close to the splenic flexure, a mark of the transition from the distal to the medial part of the colon (24).

Furthermore, the mucosa in tumoral areas was thicker in SPF Winnie mice, obscuring blood vessels visible in their littermate controls as well as in both GF Bl/6 and Winnie mice (Figure 2B, lower row). Histological analysis of 20-week-old SPF Winnie mice revealed dysplastic crypts and severe inflammation (Figure 1G, middle image plus inset), whereas CONV Winnie mice only showed diffuse inflammation (Figure 1G, upper image). Additional inflammatory features in SPF Winnie mice included crypt elongation, immune infiltration, and crypt abscesses; these features were absent in SPF Bl/6 and GF Winnie mice (Supplemental Figure 1, A–C; supplemental material available online with this article; https://doi.org/10.1172/JCI196712DS1). PAS-Alcian blue staining confirmed a general reduction of mucus-secreting cells and loss of acid mucins (Alcian blue staining) in SPF Winnie mice compared with SPF Bl/6 and GF Winnie mice (Supplemental Figure 1, D–F). Moreover, aberrant crypts exhibited a unique PAS staining pattern, characterized by a reduced number of mucus-secreting cells along the epithelium and a concentrated magenta staining in the crypt centers, indicating a shift toward neutral mucin production during tumor progression (Supplemental Figure 1, D–F). Finally, expression of several intestinal mucin mRNAs showed significant changes in both colonic tumor areas and nontumor inflamed areas in SPF Winnie mice compared with WT (Bl/6) controls (Supplemental Figure 1G). Altogether, these results underline significant alterations in mucus composition in SPF Winnie mice with colitis and CAC. Overall, our data emphasize the role of the gut microbiota in driving CAC in SPF Winnie mice, as reflected by increased inflammation, greater tumor burden, and distinct mucosal changes, highlighting the influence of environmental factors on disease progression.

### Specific microbial populations shape the fecal and tumoral phenotype of SPF Winnie mice.

**Figure 3:**
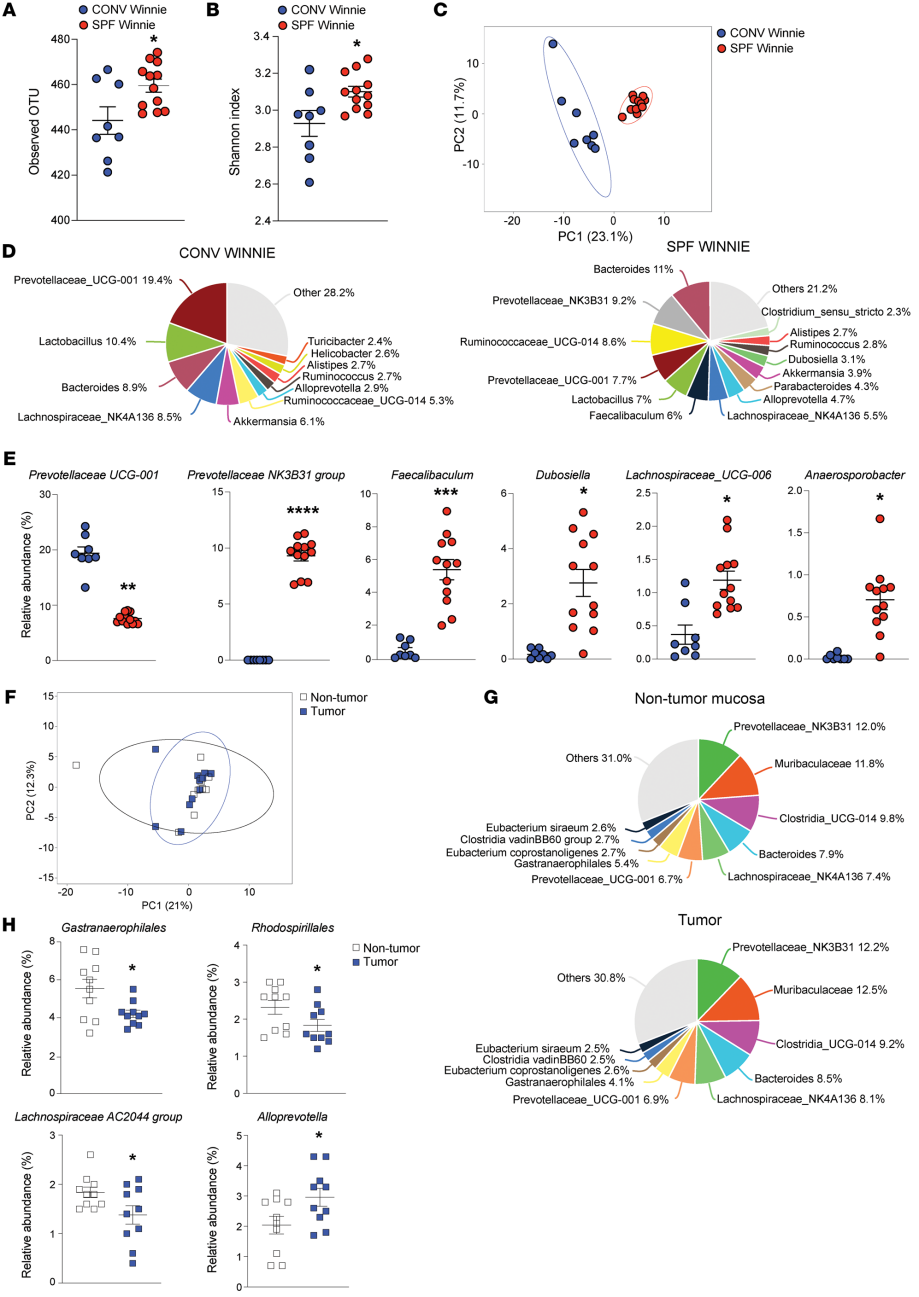
Microbiome composition differs in tumor-bearing SPF versus CONV Winnie mice, with differential regulation of *Gastranaerophilales,* *Rhodospirillales*, *Lachnospiraceae AC2044* group, and *Alloprevotella*. (**A**–**E**) 16S rRNA-Seq of fecal total microbiota from 20-week-old SPF (*n* = 12) and CONV (*n* = 8) Winnie mice. (**A**) Observed operational taxonomic unit (OTU), (**B**) Shannon diversity index, and (**C**) PCA were assessed in the 2 experimental groups. (**D**) Pie charts for SPF (right) and CONV (left) Winnie stool, indicating percentages of the most abundant genera (>1%). (**E**) Relative abundance (expressed as a percentage) of significantly represented genera in the stools of 20-week-old SPF (*n* = 12) and CONV (*n* = 8) Winnie mice. (**F**–**H**) 16S rRNA-Seq of nontumor and tumor-associated microbiota from 20-week-old SPF Winnie mice. (**F**) PCA with (**G**) pie charts depicting nontumor mucosa (upper) and tumor-associated mucosa (lower), showing percentages of the most abundant genera (>1%) and the (**H**) relative abundance (expressed as a percentage) of significantly represented genera in SPF Winnie. Data presented in the dot plots are expressed as the mean ± SEM. *n* = 12 for each experimental group. *P < 0.05, ***P < 0.0005, and ****P < 0.0001, by unpaired 2-tailed *t* test or Mann-Whitney *U* test.

Next, we performed 16S rRNA analysis to compare stool microbiota from 20-week-old SPF and CONV Winnie mice. We observed a distinct separation between SPF and CONV Winnie mice in terms of α diversity, as indicated by the observed operational taxonomic unit (OTU) and Shannon index (Figure 3, A and B, respectively), as well as β diversity (Figure 3C). The composition of the most abundant (>1%) microbial genera from SPF and CONV Winnie mice is indicated by the pie charts shown in Figure 3D. Specifically, we found significant upregulation of *Prevotellaceae_UCG-NK3B31*, *Faecalibaculum*, *Dubosiella*, *Lachnospiraceae_UCG-006*, and *Anaerosporobacter* genera in SPF versus CONV Winnie mice, while an opposite regulation was detected for the genus *Prevotellaceae_UCG-001* (Figure 3E). We also compared tumor-associated microbiota with nontumor mucosa without observing differences in β diversity (Figure 3F). Looking at the most abundant microbial genera composition in this comparison (Figure 3G), we found that 4 genera were differentially represented in SPF Winnie mice. Specifically, *Gastranaerophilales*, *Rhodospirillales*, and *Lachnospiraceae_AC2044* genera were less abundant, whereas *Alloprevotella* was more abundant in tumors compared with nontumor mucosa of SPF Winnie mice (Figure 3H). Overall, these results reveal specific bacterial genera that were differentially enriched in SPF Winnie mice, suggesting a potential link between microbiota alterations and tumor progression in these mice.

### Stool metabolomics and lipidomics highlight alter sphingolipid and nucleotide metabolism in SPF Winnie mice.

**Figure 4:**
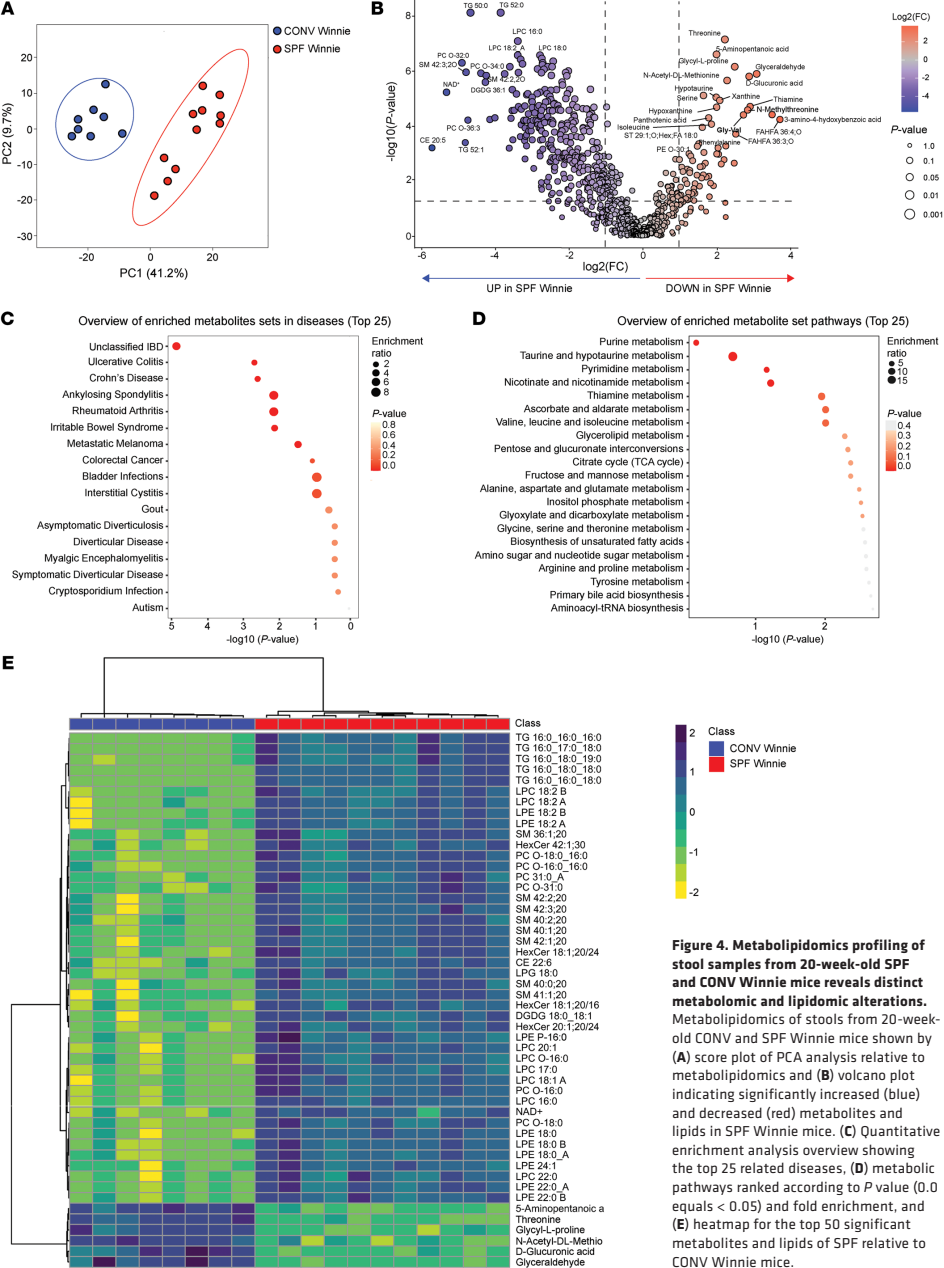
Metabolipidomics profiling of stool samples from 20-week-old SPF and CONV Winnie mice reveals distinct metabolomic and lipidomic alterations. Metabolipidomics of stools from 20-week-old CONV and SPF Winnie mice shown by (**A**) score plot of PCA analysis relative to metabolipidomics and (**B**) volcano plot indicating significantly increased (blue) and decreased (red) metabolites and lipids in SPF Winnie mice. (**C**) Quantitative enrichment analysis overview showing the top 25 related diseases, (**D**) metabolic pathways ranked according to *P* value (0.0 equals < 0.05) and fold enrichment, and (**E**) heatmap for the top 50 significant metabolites and lipids of SPF relative to CONV Winnie mice.

We conducted metabolomics and lipidomics analyses of stool samples from 20-week-old SPF and CONV Winnie mice. Principal component analysis (PCA) revealed 2 distinct clusters corresponding to the experimental groups (Figure 4A). The volcano plot identified 300 metabolites and lipids that were significantly enriched (Figure 4B, blue) and 76 that were decreased (Figure 4B, gray) in SPF mice compared with CONV Winnie mice. Enrichment analysis of the top 25 metabolites linked these metabolites to inflammatory bowel disease (IBD), inflammation, cancer, and autoimmune disorders (Figure 4C), with further associations with nucleotide metabolism and bioenergetic pathways (Figure 4D). The heatmap confirmed the distinct separation of the top 50 significant metabolites and lipids in fecal samples from the 2 groups (Figure 4E). Supplemental Figure 2 presents dot plots of some of the most significant metabolites and lipids. The dot plots in Figure 4, C and D, provide an overview of the enriched metabolite sets, where the size of the dots per metabolite set indicates the enrichment ratio, and the color intensity indicates the *P* value. Among the major lipid classes that were differentially abundant between SPF and CONV Winnie mice, sphingolipids — particularly ceramides (CerS), hexosylceramides (HexCerS), and sphingomyelins (SMs) — were the most modulated subclasses. SPF mice had higher fecal levels of SMs, HexCerS, and CerS.

Additionally, lysophosphatidylcholines (LPCs) and lysophosphatidylethanolamines (LPEs) were significantly more abundant in SPF feces (*P* < 0.0001). As for the polar metabolome, we observed a pronounced alteration in purine and pyrimidine nucleotide metabolism. The levels of adenosin-3′-monophosphate (AMP), cytidine-3′-monophosphate (CMP), guanosine-5′-monophosphate (GMP), and uridine-5-monophosphate (UMP) were all elevated in SPF mice, suggesting an upregulation of nucleotide metabolism, which is often associated with tumor growth. Consistently, we found that NAD^+^ levels were also increased in SPF mice, reflecting higher metabolic demands, as cancer cells require significantly more NAD^+^ than normal cells to sustain their elevated energy consumption.

### Shotgun metagenomic analysis reveals distinct microbial profiles and metabolic pathways in SPF and CONV Winnie mice.

**Figure 5:**
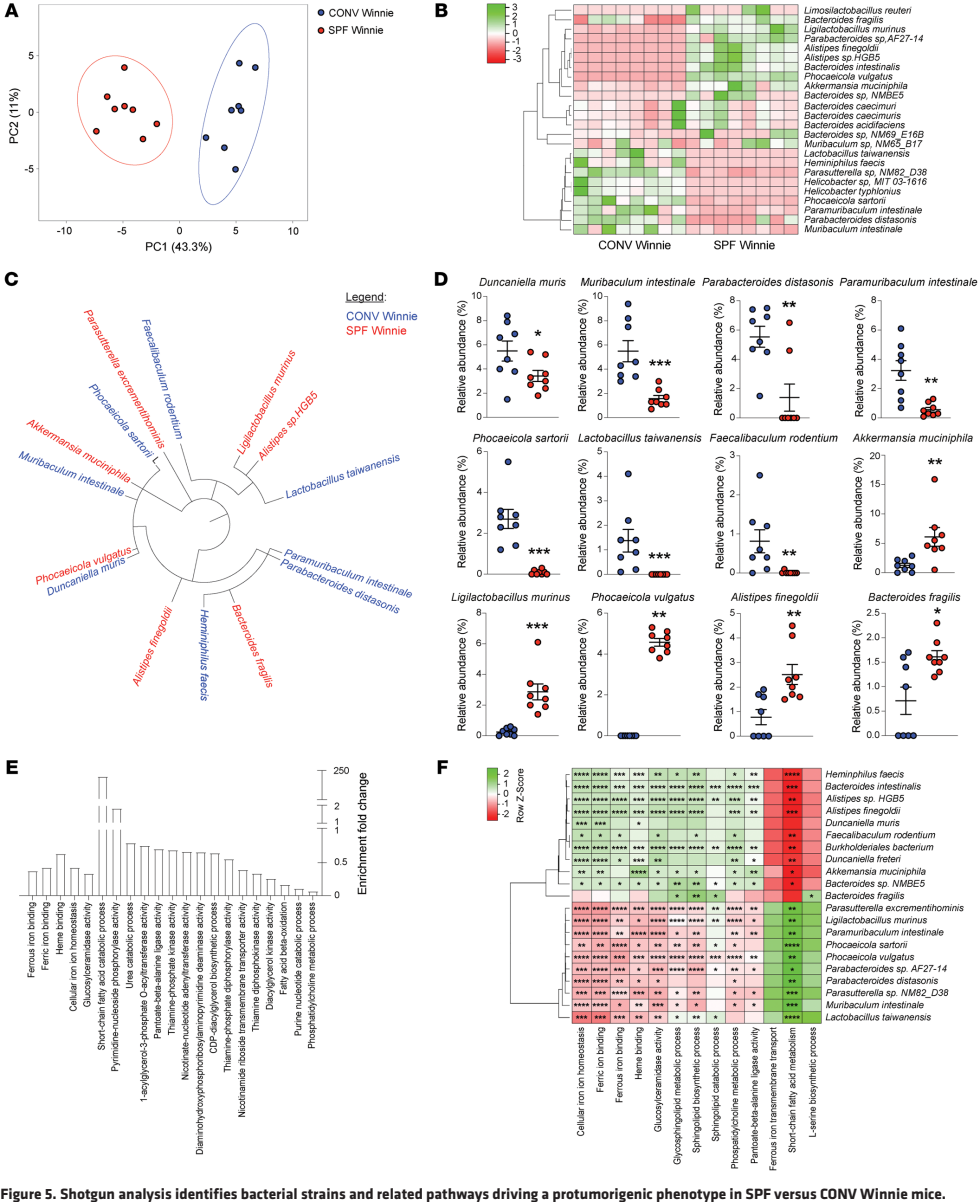
Shotgun analysis identifies bacterial strains and related pathways driving a protumorigenic phenotype in SPF versus CONV Winnie mice. Fecal samples from 20-week-old SPF versus CONV Winnie mice were analyzed and are presented by (**A**) PCA, (**B**) heatmap of the most abundant bacterial species (>1%), (**C**) circular dendrogram highlighting significantly different bacterial species, and (**D**) dot plots showing the relative abundance (expressed as a percentage) of the most abundant species. (**E**) Enrichment fold change of specific pathways relevant in colitis and colitis-associated cancer, comparing fecal material from SPF and CONV Winnie mice. (**F**) Spearman’s correlation between bacterial species and relevant pathways enriched in SPF versus CONV Winnie mice. Data presented in the dot plots are expressed as the mean ± SEM. *n* = 8 for each experimental group. *P < 0.05, **P < 0.005, ***P < 0.0005, and ****P < 0.0001 by unpaired 2-tailed *t* test or Mann-Whitney *U* test.

To build on insights from 16S sequencing, we performed shotgun sequencing of 20-week-old SPF and CONV Winnie mice. This analysis revealed a clear distinction between the 2 experimental groups, as demonstrated by the PCA score plot (Figure 5A) and microbial species clustering in the heatmap (Figure 5B). Despite these differences, we observed a close phylogenetic relationship among the most abundant and differentially represented species in SPF and CONV Winnie mice (Figure 5C). Examining the significantly regulated microbial species, we found that *Duncaniella muris*, *Muribaculum intestinale*, *Parabacteroides distasonis*, *Phocaeicola sartorii*, *Lactobacillus taiwanensis*, and *Faecalibaculum rodentium* were more abundant in CONV Winnie stool samples (Figure 5D). Interestingly, tumor-associated species such as *Alistipes finegoldii*, *Phocaeicola vulgatus*, and *Bacteroides fragilis* were enriched in SPF Winnie mice.

Additionally, in SPF Winnie mice, *Akkermansia muciniphila* and *Ligilactobacillus murinus* were significantly more abundant, even though these 2 species are frequently reported to be protective against colitis and cancer. Next, we performed a functional analysis of these bacterial species, which revealed an enrichment of proinflammatory and iron-related pathways in the SPF group (Figure 5E). These pathways correlated with the bacterial species that were more abundant in SPF Winnie mice. In contrast, SCFA metabolism was more strongly associated with bacteria characteristic of CONV Winnie mice (Figure 5F). Shotgun analysis further indicated that SPF-associated bacteria exhibited heightened iron capture and metabolic activity, with increased representation of iron ion–binding, heme-binding, and iron homeostasis pathways. Additionally, we found that several of the most prominent pathways aligned with the elevated metabolites detected in our Winnie cohorts, including those involved in thiamine, pantoate, nucleotide, and fatty acid metabolism.

### Multiomics correlation analysis reveals distinct microbial and metabolic profiles driving CAC in SPF Winnie mice.

**Figure 6:**
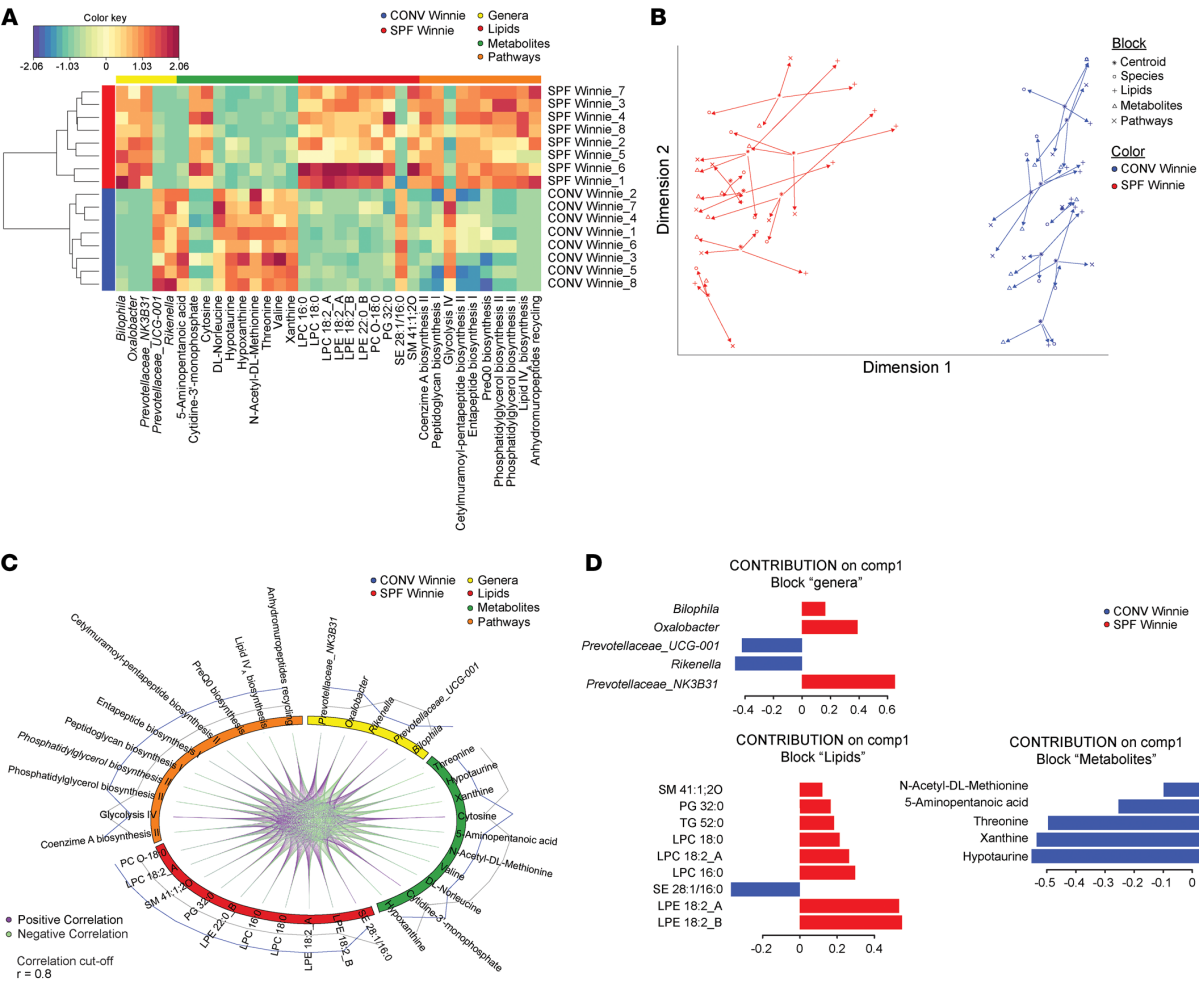
Integration of multiomics analyses shows a strong correlation between metabolomics, lipidomics, and bacterial genera. (**A**) Heatmap represents the correlation structure extracted from the 4 omics datasets. The correlation of each original feature pair is determined by each of their correlations with the components from the integrative method. (**B**) Arrow plots depict the similarities and discrepancies between a given sample across the 4 datasets, which can be seen (refer to the legend) . Short arrows indicate strong agreement between datasets, while long arrows highlight significant disagreement. Each sample is represented by a centroid and associated with 4 data types. (**C**) The circos plot depicts the correlations between each feature of each dataset. The top selected features of each dataset are shown. Lines are only drawn for correlations above 0.8 (cutoff = 0.8) to reduce visual clutter. (**D**) The loading generated from the PLS-DA applied to the multiomics data highlights the variables that discriminate between treatment conditions. Blue indicates the SPF group, and red represents the CONV group. *n* = 8 for each experimental group.

The use of the same fecal sample for 16S rRNA-Seq, metabolomics/lipidomics, and shotgun sequencing enabled direct comparisons across datasets, allowing us to uncover meaningful relationships among microbe composition, metabolic profiles, and lipidomic changes. Specifically, we performed a multiomics correlation analysis, which confirmed distinct segregation between SPF and CONV Winnie mice, consistent with patterns observed in individual analyses when all datasets were combined (Figure 6A). A 2D projection further validated this separation, reflecting the clustering trends identified in the independent analyses (Figure 6B). Focusing on dimension 1, the average distances between the principal coordinates are noticeably larger in the SPF group compared with the CONV group. Moreover, we observed strong positive correlations between differentially abundant bacterial genera, metabolites, and lipids across the groups (Figure 6C). The differential contributions of specific genera, lipids, and metabolites to the SPF and CONV Winnie phenotypes are shown in Figure 6D and suggest distinct microbial and metabolic signatures associated with each condition. This indicates a potential functional relationship between microbial composition and metabolic alterations in SPF Winnie mice. These findings offer a comprehensive, systems-level perspective on the microbial and metabolic shifts in SPF Winnie mice, underscoring the intricate interplay between the gut microbiota and host metabolism in CAC development.

### FMT from SPF Winnie, but not SPF Bl/6 mice, specifically increases the dysplasia index in GF Winnie mice.

**Figure 7:**
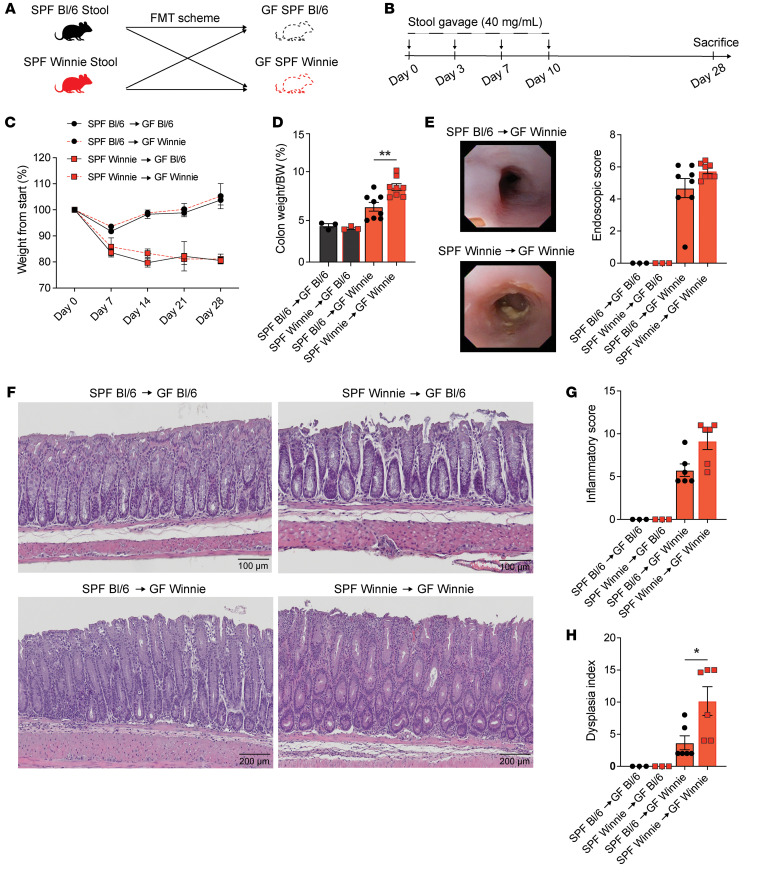
FMT using SPF Winnie and Bl/6 donors indicates that tumor development depends on Winnie, not Bl/6, recipient genotype. (**A** and **B**) FMT experimental design (**A**) and timeline (**B**) involving FMT from SPF Winnie and Bl/6 donor mice into GF Winnie and Bl/6 recipient mice. (**C**) Changes in weight of experimental mice over time and (**D**) colon weight/BW ratio (expressed as a percentage) measured at sacrifice. (**E**) Representative white light endoscopic images (left panels) of GF Winnie recipient mice receiving donor fecal microbiota from SPF Winnie (lower left panel) or SPF Bl/6 mice (upper left panel), with associated endoscopic scores (right panel). (**F**) Representative H&E-stained images of experimental groups (original magnification, ×10; scale bars: 100 μm and 200 μm), with associated histological assessment of (**G**) inflammation and (**H**) dysplasia, as evaluated by a GI pathologist blinded to the H&E-stained slides of colon samples, using an established scoring system. Data presented in the histograms are expressed as the mean ± SEM. *P < 0.05 and **P < 0.005, by ordinary 1-way ANOVA with Tukey’s multiple-comparison or Kruskal-Wallis test followed by Dunn’s test. Stool homogenates were pooled from *n* = 4 donor mice, with *n* = 3–8 recipient mice for each experimental group.

To confirm the role of microbiota in CAC onset in SPF Winnie mice, we performed FMT by transferring stool from 20-week-old SPF Winnie mice or their age-matched parental control (SPF Bl/6) into GF Winnie mice. GF Bl/6 recipient mice served as negative controls (Figure 7A). Specifically, FMT was performed by gavage (40 mg/mL) four times over 2 weeks, and mice were sacrificed 4 weeks after the first gavage (Figure 7B). Weight monitoring revealed a trend of weight reduction in recipients of SPF Winnie mouse stool, regardless of the recipient’s (GF Winnie or GF Bl/6) (Figure 7C). At sacrifice, the GF Winnie mice that received SPF Winnie stool had a higher colon weight/BW ratio compared with those transplanted with SPF Bl/6 stool (Figure 7D). Endoscopic analysis showed an increased score for GF Winnie mice transplanted with both SPF Winnie and SPF Bl/6 stools, although no significant difference was observed between these groups (Figure 7E).

Furthermore, histological analysis identified dysplastic lesions in GF Winnie mice transplanted with both SPF Bl/6 and SPF Winnie stools, although the lesions were larger in those receiving SPF Winnie stools (Figure 7F). Of note, while the colonic inflammatory score did not differ between GF Winnie mice transplanted with SPF Winnie or SPF Bl/6 stools (Figure 7G), the dysplasia index was significantly higher in GF Winnie mice transplanted with SPF Winnie stool compared with that for GF Winnie mice transplanted with stools from SPF Bl/6 (Figure 7H). In line with these results, GF Bl/6 mice receiving stool from either SPF Winnie or SPF Bl/6 mice showed no weight reduction (Figure 7C), no changes in the colon weight/BW ratio (Figure 7D), and a null score for endoscopy, inflammation and the dysplasia index (Figure 7, E, G, and H). These findings suggest that the microbiota from SPF Winnie mice played a crucial role in promoting CAC development in a genetically susceptible host.

## Discussion

Here, we describe a relevant model of early-onset CAC dependent on the gut microbiome and its metabolites. Following rederivation into an SPF facility, Winnie mice exhibited a more severe colitis phenotype and, notably, spontaneous CAC at as early as 4 weeks of age. In contrast, CONV Winnie mice developed only mild colitis as previously reported in other facilities (5), with no overt signs of tumorigenesis. We demonstrated the essential role of the gut microbiome by observing that GF Winnie mice were protected from colitis and colon tumor development. Using shotgun metagenomics, metabolomics, and lipidomics, we characterized a distinct proinflammatory microbial and metabolic signature that potentially drives the transition from colitis to CAC. FMT using either SPF Winnie or WT (Bl/6) donors into GF Winnie recipients demonstrated that, while colitis developed regardless of donor, only FMT from SPF Winnie donors resulted in CAC, revealing a critical microbiota-driven, host-specific susceptibility to tumorigenesis in this model.

Compared with CONV Winnie mice, SPF Winnie mice showed decreased BW, but increased colon weight. This was mainly due to increased inflammation and a larger tumor area. Indeed, SPF Winnie mouse colons had tumors in the proximal and medial parts, a feature resembling what is often observed in the ascending and transverse portions of patients with CAC (25, 26). Furthermore, the macroscopic features of these tumors were comparable to those of flat and serrated adenomas, which are typical of CAC (27). The dysplastic lesions had an early onset, consistent with the incidence of CAC in patients with IBD (28), unlike those with sporadic CRC or previously established models of CAC, like azoxymethane/dextran sodium sulfate (AOM/DSS) or Winnie/*APC^min/+^* mice, in which tumors appear as polyps and have a late onset in patients (29).

Microscopically, SPF Winnie mice showed increasing signs of inflammation in their colon, with dysplastic lesions appearing in 4-week-old mice. Lesions that grew slowly and steadily with age in mice, similar to those in patients with CAC (30), showed a steep increase at 20 weeks of age, concurrent with a higher dysplasia grade. On the other hand, CONV Winnie mice did not develop any tumors but only mild inflammation. Thus, differences in the microenvironment and in the housing facility led to distinct phenotypes; increased inflammation and the formation of dysplastic lesions might have been caused by the SPF facility being cleaner than the CONV one, thereby perturbing the intestinal microbiota.

One of the most striking aspects of our model is the unique microbiome composition we identified through 16S rRNA sequencing. Despite and almost identical α diversity, β diversity changed dramatically, as evidenced by the clear separation of the 2 Winnie populations on the PCA plot; moreover, several bacterial genera were differentially represented in the stools of these mice. Within the *Bacteroidetes* phylum, we noticed an opposite abundance for *Prevotellaceae-UCG001* and *Prevotellaceae NK3N31*. The first was significantly less represented in the SPF Winnie microbiota, whereas the second was absent in CONV mice and more than 9% in the SPF Winnie mice. Both groups are associated with fiber consumption and SCFA production; however, *Prevotellaceae-UCG001* has recently been linked to inhibition of colorectal cancer progression in FMT experiments (12). *Faecalibaculum*, significantly more represented in the SPF-Winnie fecal material, is a lactic acid producer with a disputed role in colon cancer. It is possible that the abundance of lactic acid in the lumen affects luminal pH, alters gut metabolism, and promotes the tumor microenvironment (31). *Faecalibaculum* has been suggested as a marker of CRC. Nonetheless, its role in SCFA production may support the idea of a prohomeostatic switch to pathogenic under particular conditions (32). Similarly, other SCFA-producing groups known for their antiinflammatory and tolerogenic potential (33–35), including *Dubosiella*, *Lachnospiraceae UCG-006*, and *Anaerosporobacter*, were increased in the SPF-Winnie fecal material. The hampered immune response may indeed accelerate tumor growth.

When we looked at the tumor-associated microbiota, we noticed that *Alloprevotella* was significantly higher in CAC tissue than the paired normal tissue of SPF Winnie, similar to what was previously reported in humans (36). *Gastranaerophilales*, *Lachnospiraceae AC2044*, and *Rhodospirillales* were reduced considerably in the healthy tissue of SPF Winnie mice, but their functional role is not fully elucidated. These bacteria may be associated with dysplastic mucosa or part of the mucosal biofilm that promotes tumor growth (37).

We performed stool metabolomics and lipidomics analyses of the same samples to gain a deeper insight into microbial activity. Similarly to what was observed for the 16S sequencing, samples from the SPF and CONV colonies separated well on the PCA plot. We identified several more abundant metabolites in SPF Winnie mice, related to energy production and amino acid recycling pathways. Among the metabolites related to nucleotide metabolism, adenosine 3′-monophosphate and adenosine 3′,5′-cyclic monophosphate were the most strongly detected in SPF Winnie, confirming what was previously reported in a fecal sample metabolomics analysis from patients with CRC (38). There is evidence that most of the metabolites we detected are produced in areas of ongoing inflammation, as immune cells utilize them to sustain their activity. Next, with stool lipidomics, we observed an interesting perturbation in the sphingomyelin/ceramide pathway. Sphingomyelin is a membrane phospholipid that can be hydrolyzed by sphingomyelinases (SMases) to generate ceramide (39). SPF Winnie mice had increased sphingomyelins in contrast to CONV mice, which had more ceramides in their stool (40). *Bacteroidetes* rise nonsignificantly in the SPF Winnie and are the only sphingolipid producers in the gut microbiome (41). Using serine palmitoyltransferase, long chain base subunit 2–KO (*Sptlc2*-KO) mice, Li et al. demonstrated that blocking the ceramide de novo synthesis pathway dramatically affects the mucus layer and E-cadherin expression in the gut, suggesting a direct link between ceramide and gut barrier function (42). LPC, which we found to be increased in SPF Winnie’s stool, is often linked to increased inflammation and CRC. Bacterial sphingolipids are indeed associated with increased inflammation and cellular division, which is connected to the onset of cancer; conversely, ceramides have been shown to exhibit antiinflammatory potential. Moreover, other metabolites we found to be increased in the stools of CONV Winnie mice, such as the sterols ST 29:1 and ST 28:1 (stigmasterol and its derivatives), are linked to decreased inflammation and protection from DSS colitis (43).

To confirm that stool bacteria produce these metabolites, we performed a functional analysis of shotgun metagenomics data. What we found was indeed a more active sphingomyelin biosynthetic pathway in SPF Winnie mouse stool compared with that of CONV mice. Furthermore, we observed a significant increase in the SCFA metabolic pathway in stools from CONV Winnie mice, indicating that some bacteria in their microbiota were actively producing SCFAs with antiinflammatory effects. Moreover, SPF bacteria exhibited increased iron metabolism, a sign often correlated with more pathogenic species, which can also increase the risk of cancer formation in IBD conditions. The presence of tumor-related and tumor-promoting bacteria was also observed with the shotgun analysis, in which species like *Alistipes finegoldii* (44) and *Bacteroides fragilis* (45–47) were more abundant in SPF Winnie mice; the same can be said for the *Phocaeicola* (48) and *Ligilactobacillus murinus* (49, 50) species, while CONV mice had increased abundance of *Duncaniella muris* and *Lactobacillus taiwanensis*, which are supposed to be protective against sustained inflammation (51, 52). Recent evidence suggests that the presence of *Akkermansia muciniphila* in the gut microbiome may be a signature of CAC susceptibility (53). Indeed, Winnie mice have increased *Akkermansia* abundance compared with their Bl/6 littermates (22), which may initiate tumorigenesis in the presence of other specific bacterial species (53, 54). The abundance of *Akkermansia muciniphila* might also indicate a more labile mucus layer that favors the epithelial exposure to luminal antigens and inflammatory mediators (54).

Using a comprehensive bioinformatics approach, we combined 16S, shotgun metagenomics, metabolomics, and lipidomics data, and this cross-analysis confirmed all the single inputs we had. Moreover, we used an integrative bioinformatics approach, which has recently been applied to characterize patients with early-onset CRC (55). We obtained a correlation signature of bacteria, metabolic pathways, and metabolites that is representative of the CONV and SPF Winnie phenotypes. Surprisingly, *Akkermansia’s* central role in cancer overlaps with our correlation. To further confirm the microbial signature of the SPF Winnie phenotype, we performed FMT experiments using GF Winnie mice. We transplanted the whole SPF Winnie stool into GF Winnie and Bl/6 recipients. GF Winnie recipients exhibited colonic inflammation, initial tumor formation, and dysplasia, whereas SPF Bl/6 microbiota mitigated this outcome. Our model stands out as a suitable recipient of FMT, exhibiting consistent microbial engraftment and replicating the donors’ phenotype well, consistent with previous results (7, 12). The stool microbiota were responsible for the formation of dysplastic lesions, which resembled those observed macroscopically and microscopically in the stool donors. Despite these encouraging results, further studies are necessary to determine whether a specific group of bacteria induces this phenotype or whether it results from a larger bacterial community. Previous results by our group lean toward the second hypothesis — that biofilm and bacteria are indeed responsible for increased intestinal inflammation — thus, the SPF tumorigenesis is likely to originate from a network of bacteria species that is absent in CONV mice and that exploits “good” bacteria metabolites and their immunosuppressive abilities to trigger intestinal inflammation and cancer onset (12, 56). Other environmental differences between the CONV and SPF facilities, such as water, diet, and light cycles, may also explain the diverse phenotypes observed in the 2 colonies.

There is a relative paucity of murine models that faithfully recapitulate CAC. The most widely used approach is the chemically induced AOM/DSS model, which relies on repeated cycles of DSS-driven epithelial injury combined with the carcinogen AOM. While highly informative, this model does not fully mimic the spontaneous development of the colitis-associated neoplasia observed in patients. By contrast, the SPF Winnie mouse model described here represents an important advance, as tumorigenesis arose in the context of a *Muc2* missense point mutation — highly relevant to human disease, in which *Muc2* variants and altered mucin biology have been reported in colitis. This genetic defect drives epithelial barrier dysfunction and chronic inflammation, with cancer progression strongly influenced by the microbiome, thereby integrating several key pathogenic processes observed in human CAC.

Importantly, neoplastic lesions in SPF Winnie mice are frequently localized to the proximal transverse colon, a site that is typically involved in inflammation and dysplasia in colitis-associated settings. The histopathologic spectrum of neoplasia also includes high-grade dysplastic lesions with a serrated morphology, a feature increasingly recognized in human CAC. Taken together, the Winnie model complements existing murine systems by providing a spontaneous, genetically driven platform that highlights epithelium-microbiome interactions, proximal colon involvement, and serrated dysplasia — features that underscore this model’s value in studying pathways highly pertinent to human CAC.

Overall, our findings reveal an intricate relationship between specific microbial communities and metabolic profiles that differed significantly from established models (9, 23, 57), suggesting that this relevant model can provide critical insights into the pathophysiology of CAC. One limitation of our study was the inability to perform cohousing experiments between CONV and SPF Winnie mice and/or to perform FMT from CONV mice into SPF Winnie mice because of specific IACUC and vivarium regulations.

In conclusion, the present data demonstrate that environmental exposure may result in CAC in susceptible individuals. These results underscore the nonredundant roles of the microbiome and metabolism in the pathogenesis of CAC. The involvement of the gut microbiome in cancer has been speculated (58, 59). Still, to the best of our knowledge, these are the first results to demonstrate a direct connection between FMT and CAC development in a mouse model that has never been reported to spontaneously develop colon cancer. The unique microbiome and metabolic profiles observed in the 2 different facilities not only enhance our understanding of disease mechanisms but also open avenues for innovative therapeutic strategies. As we continue to unravel the complexities of the microbiome-cancer nexus, our findings underscore the importance of integrating microbiological and metabolic research into cancer studies, potentially leading to more effective prevention and treatment strategies for patients with CAC.

## Methods

### Sex as a biological variable.

Our study examined male and female animals, and similar findings are reported for both sexes.

### Animal studies.

SPF and GF Winnie mice were generated from CONV Winnie mice provided by Chieppa’s laboratory by transferring preimplantation embryos into SPF or GF pseudopregnant recipient females. This technique was performed by the Case Transgenic and Targeting Core of CWRU (http://ko.cwru.edu/services/rederivation.html). Both sexes were used in all the experiments. All animals were maintained in a controlled environment (20°C–22°C, 12-hour light/12-hour dark cycles, and 45%–55% relative humidity), housed in standard cages with corn bedding, and fed a standard diet (Prolab IsoPro RMH 3000, LabDiet). Mice were sex matched for all experiments, and experiments were performed on mice aged 4, 8, 12, 16, and 20 weeks (each time point is specified throughout the text). All mice were on a C57BL/6J background (stock no. 000664, The Jackson Laboratory), and all experiments were performed on sibling littermates.

Mouse endoscopies were conducted under isoflurane anesthesia according to the IACUC protocol using a flexible uteroscope (Olympus) and following our laboratory’s standard procedure for imaging and scoring (24).

### Histology analysis.

Mice were euthanized according to the animal resource center (ARC protocols), and their colons were explanted, fixed in 10% formalin for 24 hours, and washed with and stored in 70% EtOH before paraffin embedment, sectioning, and staining with H&E or Alcian blue/periodic acid–Schiff (PAS) (Thermo Fisher Scientific) to be scored for inflammation by a trained pathologist. Colon length and weight were measured at sacrifice as indicators of colonic inflammation. The colon/BW index was calculated as a percentage, representing the ratio of colon weight to BW for each mouse.

### 16S rRNA-Seq.

Stools (180 mg) sampled from CONV and SPF 20-week-old Winnie mice were processed for DNA extraction using the QiaAMP Powerfecal Pro DNA kit (Qiagen) following the manufacturer’s protocol. For tissue samples, total DNA was extracted using the Qiagen Blood and Tissue kit and processed like stool DNA. Paired-end sequencing was carried out on the Illumina MiSeq platform by the CWRU Genomics Core facility, and the V3–V4 region was amplified for analysis of diversity inside the bacteria domain as previously done (7).

### Deep-shotgun metagenomics and functional analysis.

To better characterize the microbiota of our mice, we performed a deep-shotgun and functional analysis from the same samples used for the 16S rRNA analysis. CosmosID carried out the analysis and bioinformatics. DNA libraries were prepared from extracts using the Nextera XT DNA Library Preparation Kit (Illumina) and IDT Unique Dual Indexes with total DNA input of 1 ng. Libraries were then sequenced on an Illumina NovaSeq 6000 platform 2x150bp. Initial quality control), adapter trimming and preprocessing of metagenomic sequencing reads are done using BBduk (60). The quality-controlled reads were then translated and searched against a comprehensive, nonredundant protein sequence database, UniRef90. The mapping of metagenomic reads to gene sequences was weighted by mapping quality, coverage, and gene sequence length to estimate community-wide weighted gene family abundances as described by Franzosa et al. (61). Gene families were then annotated to MetaCyc (62) reactions (Metabolic Enzymes) to reconstruct and quantify MetaCyc metabolic pathways in the community as described by Franzosa et al. (61). Furthermore, the UniRef90 gene families were also regrouped to gene ontology (GO) terms (63) in order to get an overview of GO functions in the community.

### Metabolomics and lipidomics sample preparation.

Stool samples (200 mg) from the same animals used for the 16S sequencing were processed using the OMNImet·GUT kit (DNA Genotek) before the metabolite extraction. The samples were then processed and the metabolites were extracted as previously reported (64). Following centrifugation, polar metabolites and lipids fractions were separately collected, dried by a SpeedVac (Savant, Thermo Fisher Scientific), and stored until analysis. To assess repeatability and instrument stability over time, a QC strategy was implemented. Metabolomics and lipidomics were performed on 2 different analytical platforms (65).

### Untargeted lipidomics and metabolomics.

Lipid analysis was performed by RP-UHPLC-TIMS on a Thermo Ultimate RS 3000 coupled online to a TimsTOF Pro quadrupole time of flight (Q-TOF) system (Bruker Daltonics). Lipidomics MS data alignment, filtering, and annotation were performed with MetaboScape 2021 (Bruker). Untargeted metabolomics was carried out by hydrophilic interaction liquid chromatography-high resolution mass spectroscopy (HILIC-HRMS) on a Thermo Ultimate RS 3000 coupled to a Q-Exactive quadrupole-Orbitrap (Thermo Fisher Scientific). Metabolomics MS data alignment, filtering, and annotation were performed with MS-DIAL v4.80. Detailed conditions for liquid chromatography (LC) and mass spectrometry (MS) parameters together with all preprocessing steps are reported elsewhere (66, 67). Univariate and multivariate statistical analyses were performed with MetaboAnalyst 6.0 (https://www.metaboanalyst.ca). Data preprocessing consisted of the following steps: all metabolites or lipids missing in more than 50% of QCs and 75% of real samples were excluded, and, subsequently, data were normalized, log-transformed, and autoscaled. For missing values and zeros, the minimum value for the target molecule in the dataset was used as the replacement value. A preliminary investigation was conducted using PCA of preprocessed data. Significant (*P* < 0.05) metabolites and lipids and their human metabolome database (HMDB) codes were used to build a pathway enrichment analysis using the “enrichment” function in MetaboAnalyst.

### Integration of omics.

We used a supervised N-integration approach to jointly analyze multiomics datasets, including 16S rRNA microbial profiles, lipidomics, metabolomics, and functional pathways derived from metagenomics data, in the context of CONV and SPF Winnie mice. Principal coordinates were first extracted from each omics layer, followed by integrated partial least squares discriminant analysis (PLS-DA) using the mixOmics R package. This method constructs latent components by maximizing the covariation between datasets while optimizing class discrimination and integration. The model utilizes M-fold or leave-one-out cross-validation across a user-defined parameter grid to determine the optimal sparsity level. The resulting parameters facilitate the simultaneous selection of key discriminative features across all datasets.

### FMT.

A total of 400 mg stool from 5 male and 5 female 20-week-old SPF Bl/6 and Winnie mice was dissolved in 10 mL sterile PBS to a final concentration of 40 mg/mL. Eight-week-old germ-free Bl/6 and Winnie mice were gavaged with 200 μL of this stool suspension, twice a week for 2 weeks. Mice were monitored each day and weighed every week until sacrifice, 4 weeks after the first gavage. Mice were euthanized, and their colon was explanted, measured, and weighed. The colon was cut longitudinally, and tumor areas were measured before fixation in formalin.

### Assessment of mucin markers.

RNA extraction, reverse transcription, and quantitative real-time PCR were performed with the Aurum Total RNA Mini kit (Bio-Rad), the High-Capacity RNA-to-cDNA kit (Applied Biosystems), and LightCycler 480 SYBR Green I Master Mix (Roche), respectively. Expression of mRNA was calculated using the ΔΔCt method, and the results are presented as the fold change calculated using the formula 2^–ΔΔCt^. Mouse primers used for quantitative PCR (qPCR) are shown in Supplemental Table 1.

### Statistics.

Data analysis for dot plots and histograms was performed on GraphPad Prism 10 (GraphPad Software) using parametric and nonparametric tests, unpaired 2-tailed *t* test or Mann-Whitney *U* test, 1-way ANOVA followed by Tukey’s or Dunnett’s multiple-comparison test, or Kruskal-Wallis test followed by Dunn’s test as appropriate for group comparisons (see individual figure legends). Data were presented as mean ± SEM, with a *P* value of less than 0.05 was considered significant.

### Study approval.

Our investigations were performed under the ARRIVE guidelines, and the relevant animal protocols, which were approved by both the IACUC of the National Institute of Gastroenterology “S. de Bellis” in Italy (Organism Engaged for Compliance of Animal Wellbeing [OPBA]; DGSA protocol 768/2015-PR 27/07/2015) and the IACUC of CWRU (protocol 2014-0158) in the United States. All animal experiments performed in Italy were carried out in accordance with the national guidelines of the Italian Directive 26/2014 and approved by the Italian Animal Ethics Committee of the Ministry of Health, General Directorate of Animal Health and Veterinary Drugs.

### Data availability.

Data and analytical methods will be made available upon request to the authors. 16S rRNA gene sequencing data and shotgun metagenomics data are available in the NCBI BioProject databases (BioProject ID: PRJNA1332113; https://www.ncbi.nlm.nih.gov/bioproject/PRJNA1332113). The nontargeted metabolomics and lipidomics raw data are available in the Zenodo repository (https://doi.org/10.5281/zenodo.17153926). Values for all data points in graphs are reported in the Supporting Data Values file.

## Author contributions

GV and SDS share first authorship and contributed to the acquisition, analysis, and interpretation of data, as well as the drafting of the manuscript and statistical analysis. The authorship order was assigned using random.org. MC and FC are co–senior authors who contributed to the study concept and design, analysis and interpretation of data, drafting and critical revision of the manuscript for important intellectual content, funding acquisition, and study supervision. GV, SDS, ENM, WX, and BNI performed the experiments and analyzed the data. EMS, DL, LZ, FDAC, FM, and VC performed the computational analysis of the data. TTP and PC contributed to the drafting and critical revision of the manuscript for important intellectual content. All authors contributed to refining the study protocol and approved the final supervision.

## Funding support

This work is the result of NIH funding, in whole or in part, and is subject to the NIH Public Access Policy. Through acceptance of this federal funding, the NIH has been given a right to make the work publicly available in PubMed Central.

- NIH grants DK042191, DK055812, DK091222, DK097948 (to FC).

## Supplementary Material

Supplemental data

Supporting data values

## Version 1 11/06/2025

In-Press Preview

## Version 2 01/02/2026

Electronic publication
