# Supplementary material for: A missense mutation in Muc2 promotes gut microbiome and metabolome-dependent colitis-associated tumorigenesis
Source: J Clin Invest. 2025 Nov 6;136(1):e196712. doi: 10.1172/JCI196712 (PMC12721895; doi:10.1172/JCI196712)
Supplement: Supplemental data [file jci-136-196712-s270.pdf]

Supplemental information

A missense mutation in *Muc2* promotes gut microbiome and metabolome-dependent colitis-associated tumorigenesis.

Giulio Verna[1+], Stefania De Santis[2+], Bianca N. Islam[1], Eduardo M. Sommella[3], Danilo Licastro[4], Liangliang Zhang[5], Fabiano De Almeida Celio[1], Emily N. Miller[1], Fabrizio Merciai[3], Vicky Caponigro[3], Wei Xin[6], Pietro Campiglia[3], Theresa T. Pizarro[2], Marcello Chieppa[7*] and Fabio Cominelli[1*]

[1]Department of Medicine, Digestive Health Research Institute, Case Western Reserve University School of Medicine, Cleveland, OH, USA
[2]Department of Pathology, Case Western Reserve University School of Medicine, Cleveland, OH, USA
[3]Department of Pharmacy, University of Salerno, Fisciano, Italy
[4]AREA Science Park, Padriciano, Trieste, Italy
[5]Department of Population & Quantitative Health Sciences, Case Western Reserve University School of Medicine, Cleveland, OH, USA
[6]Department of Pathology, University of South Alabama, Mobile, AL, USA
[7]Department of Experimental Medicine (DiMeS), University of Salento, Lecce, Italy

[+]Co-first authors

*Shared senior authorship

Corresponding authors:
Prof. Fabio Cominelli, MD, PhD
Department of Medicine
Case Western Reserve University
11100 Euclid Avenue, Cleveland, 44106, OH USAPhone number: +1 216-844-7344;
fabio.cominelli@case.edu

Prof. Marcello Chieppa, PhD
Department of Experimental Medicine (DiMeS)
University of Salento
Via Monteroni 165, Lecce, 73100, Italy
Phone number: +39 083 2298869; marcello.chieppa@unisalento.it

Conflicts of interest: The authors declare no potential conflicts of interest.

Supplemental Figure 1.

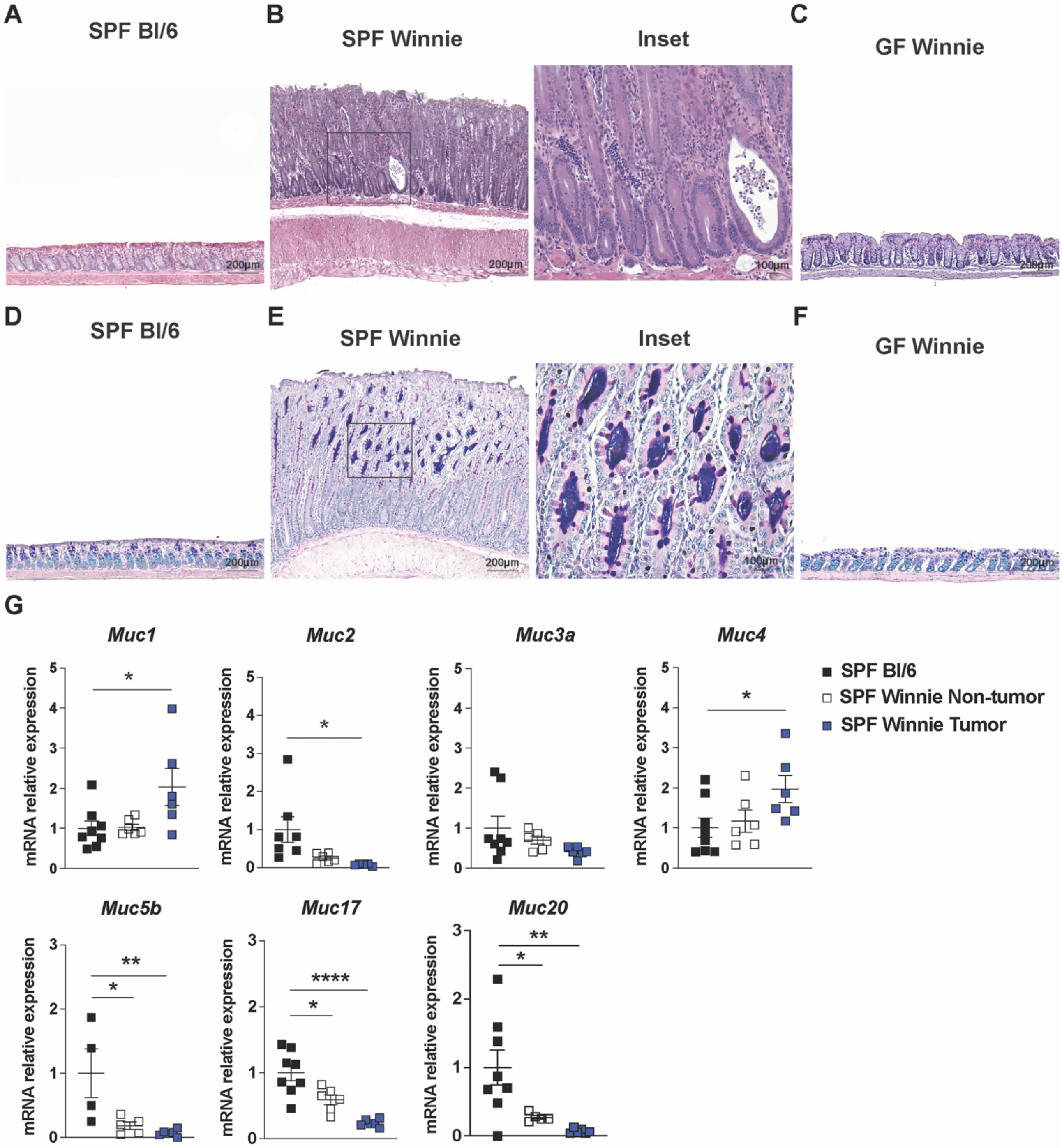

Supplemental Figure 1: Mucus composition is markedly different in SPF Winnie mice compared to Bl/6 and GF Winnie mice.

(**A**-**C**) Representative H&E images of colon tissue from 20-week-old SPF Bl/6 (**A**), SPF Winnie (**B**), and GF Winnie (**C**) mice; inset from SPF Winnie mice highlights aberrant crypts, inflammatory infiltrates, and crypt abscesses. Magnification: 5X+2, except for SPF Winnie 5X+1.65 and its inset: 20X+1.25. (**D**-**F**) Representative images of PAS/Alcian-stained colon from 20-week-old SPF Bl/6 (**D**), SPF Winnie (**E**), and GF Winnie (**F**) mice; inset highlights low staining for acid mucins (*light blue*) and an increase of neutral mucins (*magenta*) in aberrant crypts of 20-week-old SPF Winnie mice. Magnification: 5X+2, except for SPF Winnie, inset: 20X+2. **(G)** Quantification of mRNA expression for several intestinal mucins shows increased levels of *Muc1* and *Muc4* and decreased levels of *Muc2*, *Muc5b*, *Muc17*, and *Muc20* in SPF Winnie tumor areas compared to Bl/6 mice. In addition,

Muc5b, Muc17, and *Muc20* were also downregulated in SPF Winnie non-tumor areas compared to controls. Data presented in the dot plots are expressed as mean ± SEM, with *P*-values calculated by ordinary one-way ANOVA followed by Dunnett's test; *n*=5-6 (SPF mice), *n*=4-8 (Bl/6); **P*<0.05; ***P*<0.01; *****P*<0.0001.

Supplemental Figure 2.

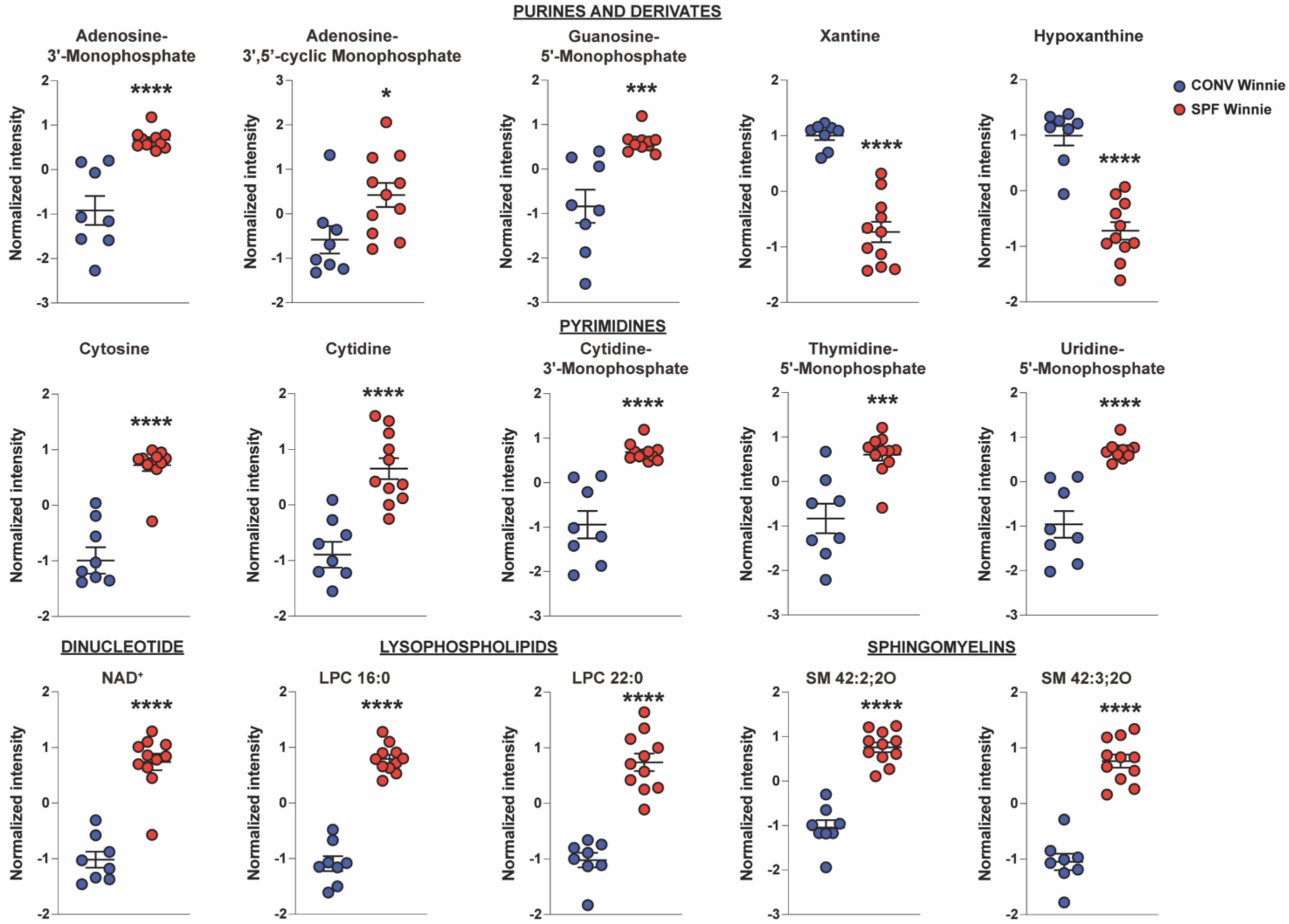

Supplemental Figure 2: Metabolipidomic profiles differ in tumor-bearing SPF vs. CONV Winnie mice, with alterations in purine and derivatives, pyrimidines, dinucleotide, lysophospholipids, and sphingomyelins.

Dot plots for normalized intensity of highly significant metabolites and lipids found in 20-week-old SPF (*red*) vs. CONV (*blue*) Winnie stool divided by compound class, *i.e.*, purine and derivatives, pyrimidines, dinucleotide, lysophospholipids, sphingomyelins. Data presented in the dot plots are expressed as mean ± SEM, with *P*-values calculated by unpaired *t*-test or Mann-Whitney; *n*=12 (SPF mice), *n*=8 (CONV mice); **P*<0.05; ****P*<0.0005; *****P*<0.0001.

Supplemental Table 1. Mouse Primers used for qPCR

Mouse Gene name	Forward 5’ → 3’	Reverse 5’ → 3’
Actb	CACTGTCGAGTCGCGTCC	TCATCCATGGCGAACTGGTG
Muc1	CCACACTCACGGACGCTAC	CATTACCTGCCGAAACCTCCTC
Muc2	CCGGGAAATGCTGTCCAGTT	TGGAGATCCGGGCTGGTATT
Muc3a	GGAAATCCTGTCCTTGAGGAGG	CCTGACAGCTGTTGCCATCT
Muc4	GCCCGCTCATCCACTATCTG	CTCCAGCATACTTAGGTTCAGAGC
Muc5b	AGTCAGAGAGTGTATAGTGACTCCA	GGTGTAAGGCGCTCATGCTA
Muc17	CGGAAGTGTGTGGGATGGAG	CTGTTTTCCCCGTCTGTGGTT
Muc20	CTTGTACGGCTGACCGTGG	TTCACGGCGGAGCTGGTTC
